# Treatment outcomes of combination versus monotherapy in *Stenotrophomonas maltophilia* bacteremia: a retrospective single-center analysis

**DOI:** 10.1128/aac.01297-25

**Published:** 2026-04-30

**Authors:** Ming-Hang Tsai, Wei-Ping Chen, Chien-Liang Chen, L. Kristopher Siu, Ching-Mei Yu, Hung-Sheng Shang, Feng-Yee Chang, Jung-Chung Lin, Ya-Sung Yang, Ching-Hsun Wang

**Affiliations:** 1 Division of Infectious, Department of Medicine, Tri-Service General Hospital SongShan Branch, National Defense Medical University 71548 , Taipei, Taiwan; 2 Division of Infectious Diseases, Department of Internal Medicine, Taoyuan Armed Forces General Hospital 90989, Taoyuan, Taiwan; 3 Division of Infectious Diseases, Department of Internal Medicine, Zuoying Armed Forces General Hospital 46674, Kaohsiung, Taiwan; 4 Institute of Infectious Diseases and Vaccinology, National Health Research Institutes, Miaoli, Taiwan; 5 Department of Pathology, Tri-Service General Hospital, National Defense Medical University 71548 , Taipei, Taiwan; 6 Division of Infectious Diseases and Tropical Medicine, Department of Medicine, Tri-Service General Hospital, National Defense Medical University 71548 , Taipei, Taiwan; University of California San Francisco, San Francisco, California, USA

**Keywords:** *Stenotrophomonas maltophilia*, bacteremia, combination therapy, monotherapy, mortality, adverse drug reactions

## Abstract

A combination therapy (CT) is recommended for *S. maltophilia* infections; however, supporting clinical data are limited. This study aimed to compare the effectiveness and safety of CT and monotherapy (MT) in the treatment of *S. maltophilia* bacteremia. A total of 292 adult inpatients with monomicrobial *S. maltophilia* bacteremia who received *in vitro* active CT or MT for >48 h were included for comparison. Multivariable logistic regression analysis was performed. The primary outcome was 30-day mortality, and the secondary outcomes included in-hospital mortality, microbiological eradication rate, clinical response, recurrent bacteremia, isolation of non-susceptible *S. maltophilia* isolates, and adverse drug reaction (ADR) occurrence. Thirty-seven patients received CT, and 255 received MT. Overall, the 30-day mortality was 24.0%, and patients treated with CT had lower 30-day mortality than those with MT (10.8% vs 25.9%; *P* = 0.045). The multivariable regression analysis revealed that CT was an independent protective factor. In addition, sensitivity analyses excluding patients with bacterial co-infections showed a consistent trend. Other outcomes were similar between the groups: in-hospital mortality (37.8% vs 36.9%, *P* = 0.909), clinical response (73.0% vs 71.8%, *P* = 0.879), microbiological eradication rate (94.6% vs 93.0%, *P* = 1.000), recurrent bacteremia (0% vs 9.4%, *P* = 0.087), non-susceptible *S. maltophilia* isolation (24.3% vs 14.9%, *P* = 0.145), and ADR occurrence (8.1% vs 13.7%, *P* = 0.441). CT for *S. maltophilia bacteremia* was associated with improved outcomes and comparable rates of ADRs and resistance emergence; however, randomized trials with larger cohorts are warranted to confirm these findings.

## INTRODUCTION

*Stenotrophomonas maltophilia* is a non-fermenting, aerobic, Gram-negative bacillus commonly found in water-associated environments, both within and outside healthcare settings (1). Despite its relatively low virulence, *S. maltophilia* has emerged in recent years as an important opportunistic pathogen that can cause severe infections in vulnerable populations in both the community and healthcare environments (2). Among the various clinical presentations of *S. maltophilia* infection, pneumonia and bacteremia are the most common and are associated with high mortality rates reported to range from 18% to 69% (2, 3). Optimal treatment options for *S. maltophilia* are limited owing to its multidrug-resistant phenotype driven by both intrinsic and acquired resistance mechanisms, restricted availability of reliable susceptibility testing, and insufficient pharmacokinetic/pharmacodynamic data for accurate clinical breakpoint interpretation (4). Recent pharmacodynamic studies have shown that several antibiotics with *in vitro* activity against susceptible *S. maltophilia* isolates exhibit limited antimicrobial efficacy at the current susceptibility breakpoints, whereas combination antibiotic regimens demonstrate enhanced bactericidal activity (5–9). In light of these findings, the Infectious Diseases Society of America (IDSA) and the Spanish Society of Infectious Diseases and Clinical Microbiology (SEIMC) recommended combination therapy (CT) with at least two active agents for the treatment of *S. maltophilia* infections (10, 11). Nevertheless, the superior *in vitro* antimicrobial activity of CT has not consistently translated into improved clinical outcomes. Several studies conducted in patients with pneumonia have shown no clinical benefit of CT over MT among overall patient cohorts (12–15). One study focused on deep seated *S. maltophilia* infections showed levofloxacin-based combination therapies were associated with reduced odds of clinical failure compared to monotherapy (16). In bacteremia, another severe manifestation of *S. maltophilia* infection, the potential clinical benefits of CT have not been clearly demonstrated (17–21). Moreover, the safety outcomes of CT and MT have not been well studied. Therefore, this study aimed to compare the clinical efficacy and safety of CT and MT for the treatment of *S. maltophilia* bacteremia.

## MATERIALS AND METHODS

### Study design and population

This retrospective cohort study was conducted at the Tri-Service General Hospital, a 1,800-bed tertiary care center in northern Taiwan, and was approved by the Institutional Review Board (2-101-05-074). Adult inpatients (>18 years) with a first episode of S. *maltophilia* monomicrobial bacteremia between January 2004 and June 2025 were identified via the microbiology database. Each identified patient was assigned a unique case number for subsequent analyses.

The final evaluable cohort comprised patients with *Stenotrophomonas maltophilia* bacteremia who received at least 48 h of *in vitro* active antimicrobial therapy administered either intravenously or orally (trimethoprim/sulfamethoxazole [SXT], levofloxacin, or minocycline). Patients were excluded if they had polymicrobial bacteremia, did not receive *in vitro* active antimicrobial therapy (SXT, levofloxacin, or minocycline), or died within 48 h after the initiation of *in vitro* active therapy. Patients were classified as receiving monotherapy (MT; a single *in vitro* active agent, including trimethoprim/sulfamethoxazole, levofloxacin, or minocycline) or combination therapy (CT; two or more active agents, including trimethoprim/sulfamethoxazole plus levofloxacin, levofloxacin plus minocycline, trimethoprim/sulfamethoxazole plus minocycline, or all three agents). In cases where both MT and CT regimens were administered during the treatment course, patients were classified according to the regimen used for the longer duration, which was defined as the index regimen.

### Data collection and definitions

Demographic data, comorbidities, source of infection, clinical status, treatment, and outcomes were extracted from the hospital’s electronic medical records (EMR) system. This system has maintained comprehensive inpatient clinical records since 2004. Data extraction and review were independently conducted by three investigators (W.-P.C., M.-H.T., and J.-C.L.). Hospital-acquired infection was defined as bacteremia ≥48 h after admission. Comorbid conditions documented at the time of admission were recorded, and the overall comorbidity burden was assessed using the Charlson Comorbidity Index (22). The source of bacteremia was identified according to the National Healthcare Safety Network (NHSN) surveillance definitions (23). Cases lacking an identifiable infectious focus were categorized as having an unidentified source of infection. The severity of infection at the onset of bacteremia was assessed using the Acute Physiology and Chronic Health Evaluation II (APACHE II) score. Acute kidney injury was defined as stage 2 and 3 based on KDIGO (Kidney Disease: Improving Global Outcomes) classification (24). Septic shock was defined according to previously established criteria (25). Concurrent infections caused by other bacteria or fungi within 48 h of the onset of *S. maltophilia* bacteremia were documented as co-infections. Patients with bacterial infections at distinct anatomical sites were recorded according to the NHSN surveillance definitions as corroborated by the growth of pathogens in the corresponding appropriate culture specimens (23). Only proven invasive fungal co-infections were documented based on the European Organization for Research and Treatment of Cancer/Mycoses Study Group Education and Research Consortium (EORTC/MSGERC) consensus definitions since this category can apply to any patient regardless of whether the patient is immunocompromised (26). Two senior infectious disease specialists (F.-Y.C. and J.-C.L.) reviewed each case to differentiate between infection and colonization and identified proven invasive fungal infections from them according to EORTC/MSGERC criteria. Immediate effective antibiotic use for *S. maltophilia* was deemed appropriate if at least one active agent against *S. maltophilia* was administered within 48 h of bacteremia onset. Appropriate antibiotic dosing for *S. maltophilia* bacteremia followed the Sanford Guide to Antimicrobial Therapy for *S. maltophilia* infections (27). For combination therapy, appropriate dosing was defined as the administration of all antibiotics targeting *S. maltophilia* within the recommended dosage ranges. Infection source control was defined as the removal of a pre-existing central venous catheter or drainage of a localized abscess presumed to be the infection source, performed after the onset of bacteremia.

The primary study outcome was all-cause 30-day mortality. Secondary outcomes were in-hospital mortality, length of hospital stay (LOS) after bacteremia among survivors, clinical response, microbiological eradication, recurrent *S. maltophilia* bacteremia*,* non-susceptible *S. maltophilia* isolation, and antibiotic-related adverse reactions (ADRs). Clinical response was assessed at the end of therapy (EOT) by two senior infectious disease specialists (F.-Y.C. and J.-C.L.) and was defined as the improvement of all signs and symptoms of infection, together with the normalization or improvement of relevant inflammatory biomarkers (C-reactive protein [CRP] or procalcitonin [PCT] levels). Microbiological eradication was defined as the absence of *S. maltophilia* in the follow-up cultures before and after EOT. Patients without follow-up blood culture results were excluded from the analysis. Recurrent bacteremia was defined as a positive blood culture for *S. maltophilia* within 30 days after EOT in patients with prior eradication. The emergence of non-susceptible *S. maltophilia* was defined as the isolation of *S. maltophilia* that demonstrated non-susceptibility to previously administered antibiotics from any clinical specimen during treatment or within 30 days after EOT. The patients were also evaluated for ADRs related to the drug administered by two investigators (W.-P.C. and J.-C.L.) using the Naranjo Adverse Drug Reaction Probability Scale, as described in our previous study (28).

### Microbiological testing

During the study period from 2004 to 2015, pathogen identification was performed using a VITEK 2 automated system (bioMérieux, Marcy l’Etoile, France). Since 2016, matrix-assisted laser desorption/ionization time-of-flight mass spectrometry (MALDI-TOF MS), specifically the VITEK MS system (bioMérieux, Marcy l’Etoile, France), has been adopted for microbial identification. Antimicrobial susceptibility testing of the cultured pathogens was conducted using a VITEK 2 system. For *S. maltophilia*, susceptibility testing included levofloxacin, SXT, and minocycline susceptibility testing (the disk diffusion method), which began in 2022. All susceptibility interpretations were performed according to the 2024 Clinical and Laboratory Standards Institute guidelines. Carbapenem-resistant organisms were defined as those resistant to at least one carbapenem.

### Statistical analyses

Continuous variables were reported as median (interquartile range) and categorical variables as counts (%). Normality was assessed using the Kolmogorov-Smirnov test. Between-group comparisons were performed using the Student’s *t*-test or Mann-Whitney *U* test for continuous variables and Chi-square or Fisher’s exact test for categorical variables. Logistic regression identified factors associated with 30-day mortality; variables with *P* ≤ 0.1 in univariable analysis and deemed clinically relevant were, thus, included in a multivariable logistic regression analysis (3, 29). Because comparison of antimicrobial therapy regimens was the primary aim of the current study, variables related to antimicrobial therapy—including combination therapy, appropriate dosage, and timing of administration—were included in the multivariable regression model regardless of statistical significance. Results were expressed as odds ratios (ORs) with 95% confidence intervals (*CIs*). Kaplan–Meier survival curves were compared using the log-rank test. We conducted several additional sensitivity analyses. First, patients in the MT group who concurrently received tigecycline, colistin, or quinolones other than levofloxacin were excluded to minimize potential confounding effects of these antimicrobial agents in the MT group. Second, patients with co-infections by other bacteria were excluded, as the presence of additional pathogens could influence outcome analyses. The remaining subgroups after exclusion were reanalyzed to evaluate the impact of CT on 30-day mortality. Statistical analysis was performed using SPSS v27.0 (SPSS Inc., Chicago, IL, USA); *P* <0.05 was considered statistically significant.

## RESULTS

### Study population

**Fig 1:**
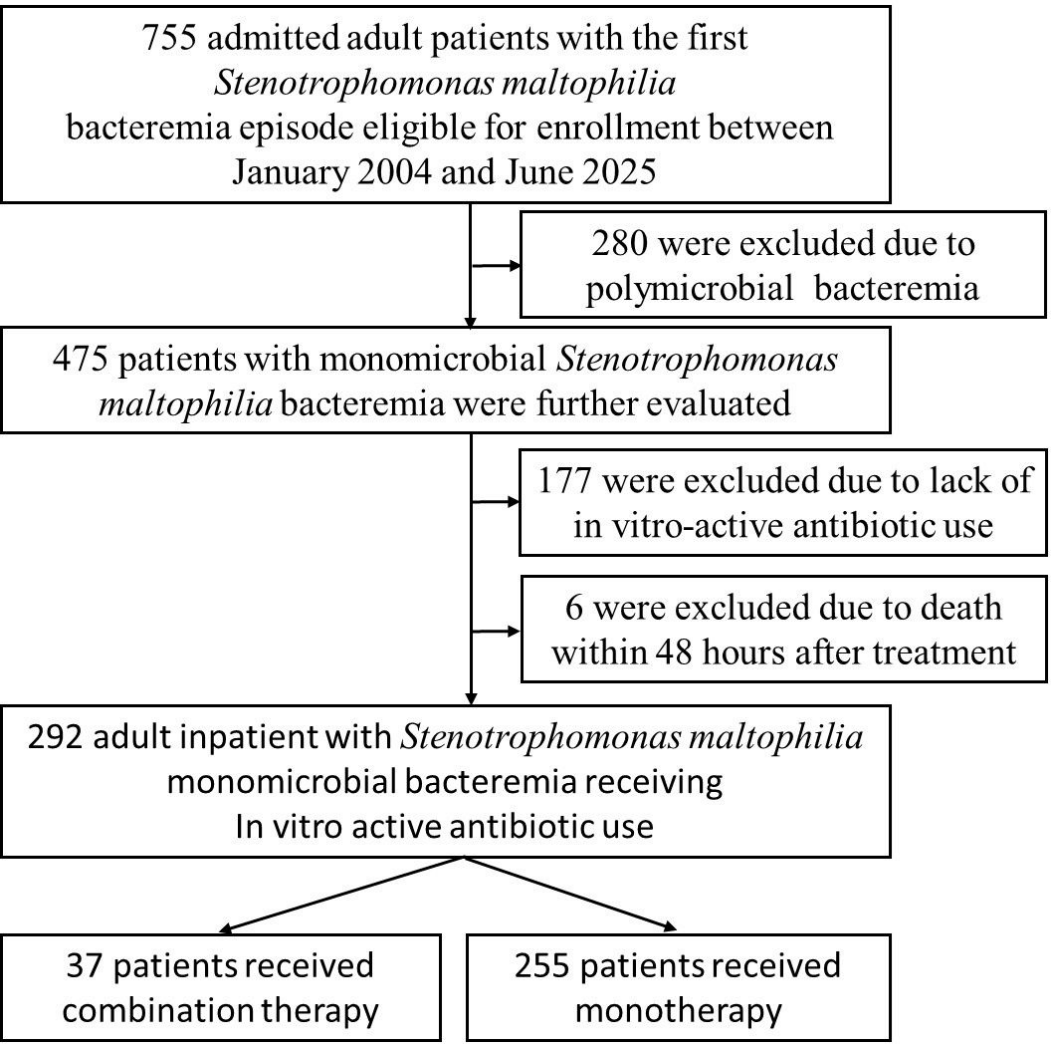
Flowchart of patients’ inclusion criteria for *Stenotrophomonas maltophilia* bacteremia treated with combination vs monotherapy.

During the study period, 755 adult inpatients with a first episode of *S. maltophilia* bacteremia were initially identified, of whom 292 met the inclusion criteria. Among the corresponding 292 *S. maltophilia* isolates, non-susceptibility to SXT was observed in 0% (0/22), 1.4% (1/71), 6.8% (8/117), and 12.1% (10/82) of isolates during 2004–2009, 2010–2014, 2015–2019, and 2020–2025, respectively. Non-susceptibility to levofloxacin was 4.5% (1/22), 5.6% (4/71), 4.2% (5/117), and 10.9% (9/82) across the same periods. An increasing trend in resistance to both agents was observed over time (Fig. S1). The patients were categorized into two groups: 37 patients who received CT and 255 who received MT (Fig. 1). Baseline characteristics were comparable between the groups in terms of demographics, comorbidities, bacteremia sources, and clinical status at onset (Table 1). A total of 136 co-infection episodes caused by bacteria other than *S. maltophilia* were identified in 117 patients (Table S1), with the causative pathogens listed in Table S2. Forty patients had co-infections with carbapenem-resistant organisms, with no significant differences between the groups; details are shown in Table S3. Patients in the CT group were more likely to receive immediate effective antimicrobial therapy than those in the MT group (40.5% vs 16.5%, *P* <0.001). The most common regimen in the CT group was SXT plus levofloxacin (26/37, 70.2%), whereas levofloxacin MT predominated in the MT group. Details of regimen changes in both groups were summarized in Table 1. Only 123 patients (42.1%) received the appropriate antibiotic dose for *S. maltophilia*, with appropriate dosing more frequent in the MT group (45.1% vs 21.6%, *P* = 0.007). Treatment duration was longer in the CT group than in the MT group (20 vs 14 days, *P* <0.001). Concurrently administered antibiotics for coinfections (excluding those targeting *S. maltophilia*) are summarized in Table S4, with no significant differences between the groups.

**TABLE 1:** Clinical characteristic comparisons among patients with *S. maltophilia* bacteremia who received CT vs MT^*e*^

Characteristics	Total (*n* = 292)	CT (*n* = 37)	MT (*n* = 255)	*P* value
Demographics, *n* (%)				
Age, years^*a*^	71.5 (59–80.75)	71 (61–79.5)	72 (58–81)	0.618
Male sex	194 (66.4)	20 (54.1)	174 (68.3)	0.088
Body weight (kg)^*a*^	59.75 (51–70)	60 (51.5–70)	59 (50–70)	0.840
Hospital acquired	286 (97.9)	37 (100)	249 (97.6)	1.000
Hospital LOS before bacteremia (day)^*a*^	23 (13–38.75)	25 (15–45.5)	23 (13–38)	0.357
ICU admission at bacteremia onset	211 (72.3)	26 (70.3)	185 (72.5)	0.844
Comorbidities, *n* (%)				
Heart failure	68 (23.3)	9 (24.3)	59 (23.1)	0.873
Old cerebrovascular accident	47 (16.1)	6 (16.2)	41 (16.1)	0.983
Chronic obstructive pulmonary disease	23 (7.9)	2 (5.4)	21 (8.2)	0.750
Diabetes mellitus	103 (35.3)	15 (40.5)	88 (34.5)	0.473
Liver cirrhosis	22 (7.5)	2 (5.4)	20 (7.8)	1.000
Chronic kidney disease^*b*^	63 (21.6)	11 (29.7)	52 (20.4)	0.197
Malignancy	94 (32.2)	13 (35.1)	81 (31.8)	0.682
Autoimmune disease	12 (4.1)	1 (2.7)	11 (4.3)	1.000
Charlson Comorbidity Index^*a*^	3 (2–5)	3 (0.5–4.5)	3 (2–5)	0.671
Source sites of bacteremia, *n* (%)				0.839
Respiratory tract	143 (49.0)	19 (51.4)	124 (48.6)	
Central line associated	85 (29.1)	12 (32.4)	73 (28.8)	
Unidentified	54 (18.5)	5 (13.5)	49 (19.2)	
Others^*c*^	10 (3.4)	1 (2.7)	9 (3.5)	
Clinical conditions at bacteremia onset, *n* (%)				
APACHE II score^*a*^	19 (14–24)	19 (13–25.5)	19 (14–24)	0.971
Septic shock	62 (21.2)	7 (18.9)	55 (21.5)	0.713
Thrombocytopenia^*d*^	76 (26.0)	12 (32.4)	64 (25.1)	0.342
Acute kidney injury	57 (19.5)	6 (16.2)	51 (20.0)	0.587
Concurrent invasive fungal infection	1 (0.3)	0 (0)	1 (0.4)	0.703
Coinfections by other bacterial species	117 (40.1)	15 (40.5)	102 (40.0)	0.950
Coinfections by other carbapenem-resistant organisms	69 (23.6)	9 (24.3)	60 (23.5)	0.915
Antibiotic treatment				
Immediate effective antibiotic targeting *S. maltophilia*	57 (19.5)	15 (40.5)	42 (16.5)	<0.001
Different antibiotic regimens targeting for *S. maltophilia*				
SXT + levofloxacin		26	–^*f*^	
SXT + minocycline		3	–	
Levofloxacin + minocycline		4	–	
SXT + levofloxacin + minocycline		1	–	
Levofloxacin + minocycline followed by SXT + minocycline		2	–	
SXT + minocycline followed by minocycline + levofloxacin		1	–	
Levofloxacin only		–	129	
SXT only		–	97	
Minocycline only		–	8	
Levofloxacin followed by minocycline		–	3	
SXT followed by levofloxacin		–	10	
Levofloxacin followed by SXT		–	8	
Appropriate antibiotic treatment dosage for *S. maltophilia*	123 (42.1)	8 (21.6)	115 (45.1)	0.007
Treatment duration, day	14 (9–18.75)	20 (14–20.5)	14 (8–17)	<0.001
Source control measures	58 (19.9)	8 (21.6)	50 (19.6)	0.774

^*a*^ Data are presented as median (interquartile range).

^*b*^ Blood creatinine levels above 3 mg/dL.

^*c*^ One is from wound culture, three from intra-abdominal sites, and six from the urinary tract.

^*d*^ Platelet count of less than 150,000/µL.

^*e*^ CT, combination therapy; MT, monotherapy; LOS, length of stay; ICU, intensive care unit; APACHE, Acute Physiology and Chronic Health Evaluation; SXT, trimethoprim/sulfamethoxazole.

^*f*^ –, not applicable.

### Primary outcomes

**Fig 2:**
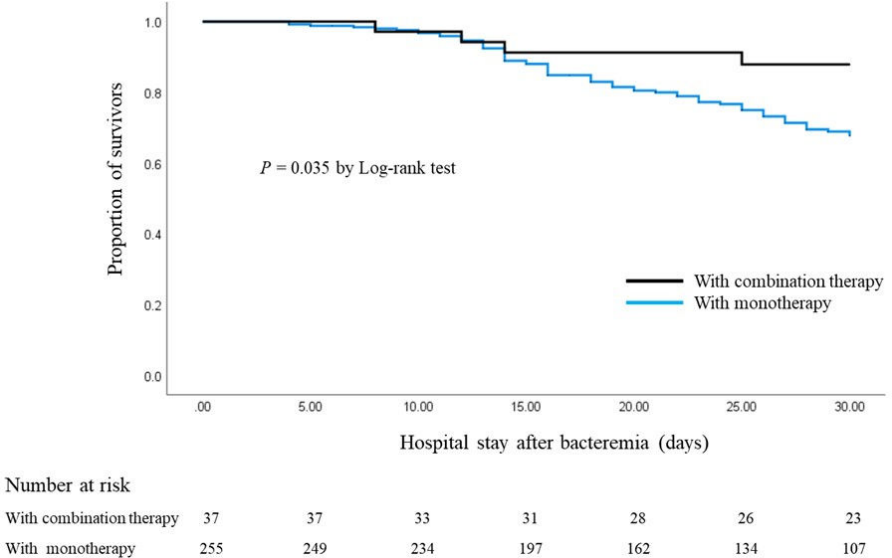
Kaplan–Meier plot in patients with *Stenotrophomonas maltophilia* bacteremia receiving combination therapy and monotherapy.

**Fig 3:**
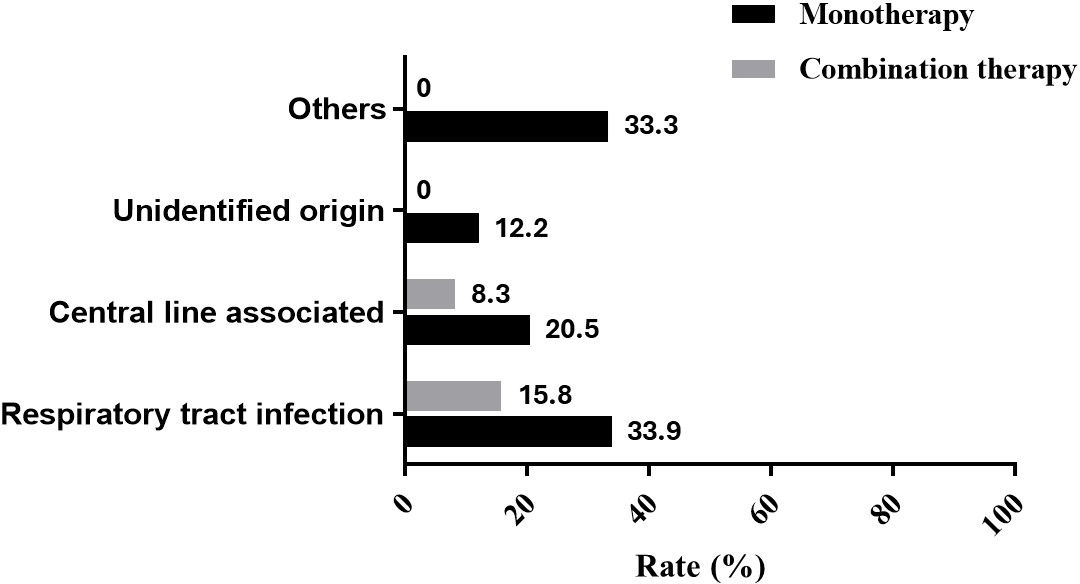
Thirty-day mortality in patients with *Stenotrophomonas maltophilia* bacteremia of different infection source sites between combination therapy and monotherapy.

Patients receiving CT had lower 30-day mortality than those receiving MT (10.8% vs 25.9%, *P* = 0.045), consistent with Kaplan–Meier analysis (*P* = 0.035; Fig. 2). Mortality by infection source is shown in Fig. 3 including respiratory tract (33.9% vs 15.8%), central line associated (20.5% vs 8.3%), unidentified (12.2% vs 0%), and other types of infections (33.3% vs 0%) for MT vs CT, respectively. After excluding patients in the MT group who had received concomitant potentially *in vitro* active agents without available susceptibility data (MT group, *n* = 204; CT group, *n* = 37), CT again demonstrated a numerically lower 30-day mortality although the difference was no longer statistically significant (10.8% vs 25.5%, *P* = 0.052). After excluding patients with co-infections caused by other bacteria (MT group, *n* = 153; CT group, *n* = 22), reanalysis showed lower 30-day mortality in the CT group compared with the MT group; however, the difference did not reach statistical significance (13.6% vs 28.8%, *P* = 0.135). Because *Acinetobacter* spp. was the most common co-pathogen in our cohort, an additional analysis excluding patients with concomitant *Acinetobacter* spp. infection was performed to minimize potential confounding (MT group, *n* = 208; CT group, *n* = 30). Consistently, the CT group remained associated with lower 30-day mortality although statistical significance was not achieved (13.3% vs 26.9%, *P* = 0.109). Univariable analysis identified respiratory tract infections (OR 2.278, 95% CI, 1.306–3.972; *P* = 0.004), unidentified origin (OR 0.331, 95% CI, 0.135–0.810; *P* = 0.015), APACHE II score (OR 1.127, 95% CI, 1.077–1.179; *P* <0.001), and thrombocytopenia (OR 3.872, 95% CI, 2.170–6.910; *P* < 0.001) as significant factors for 30-day mortality. CT (OR 0.347, 95% CI 0.118–1.017; *P* = 0.054) and source control measures (OR 0.444, 95% CI 0.199–0.989; *P* = 0.057) demonstrated a trend for improved survival, yet neither were statistically significant. In the multivariable analysis, CT was a protective factor against mortality (aOR 0.229, 95% CI, 0.070–0.750; *P* = 0.015). In contrast, a higher APACHE II score (aOR 1.101, 95% CI, 1.048–1.156; *P* <0.001) and thrombocytopenia (aOR 2.708, 95% CI, 1.438–5.102; *P* = 0.002) were independently associated with increased mortality (Table 2). Sensitivity analyses using multivariable logistic regression analyses yielded consistent findings across models (Tables S5 to S7), with CT remaining associated with lower mortality after adjustment.

**TABLE 2:** Variables associated with 30-day mortality^*a*^

Variable	Survivors (*n* = 222)	Nonsurvivors (*n* = 70)	Univariable		Multivariable	
			Unadjusted OR (95% CI)	*P* value	Adjusted OR (95% CI)	*P* value
Demographics and comorbidities						
Age	73.5 (59–80)	73.5 (58–83.25)	1.010 (0.993–1.027)	0.264		
Male sex	147 (66.2)	47 (67.1)	1.043 (0.589–1.846)	0.886		
Charlson Comorbidity Index	3 (2–5)	3 (2–5)	1.004 (0.897–1.124)	0.946		
Hospital acquired	218 (98.2)	68 (97.1)	0.624 (0.112–3.481)	0.591		
Source sites of bacteremia						
Respiratory tract	98 (44.1)	45 (64.3)	2.278 (1.306–3.972)	0.004	1.146 (0.481–2.734)	0.758
Central line associated	69 (31.1)	16 (22.9)	0.657 (0.351–1.229)	0.189		
Unidentified origin	49 (22.1)	6 (8.6)	0.331 (0.135–0.810)	0.015	0.562 (0.167–1.899)	0.354
Clinical conditions at bacteremia onset						
APACHE II score	17.5 (13–22.5)	23 (19.75–27)	1.127 (1.077–1.179)	<0.001	1.101 (1.048–1.156)	<0.001
Thrombocytopenia	41 (18.7)	33 (47.1)	3.872 (2.170–6.910)	<0.001	2.708 (1.438–5.102)	0.002
Co-infection by other bacteria	94 (42.3)	23 (32.9)	0.666 (0.379–1.173)	0.159		
Co-infections by other carbapenem- resistant organisms	52 (23.4)	17 (24.3)	1.049 (0/559–1.966)	0.882		
Antibiotic treatment						
Immediate effective antibiotic targeting for *S. maltophilia*	41 (18.5)	16 (22.9)	1.308 (0.681–2.513)	0.420	1.229 (0.581–2.598)	0.590
Combination therapy for *S. maltophilia*	33 (14.9)	4 (5.7)	0.347 (0.118–1.017)	0.054	0.229 (0.070–0.750)	0.015
Appropriate antibiotic treatment dosage for *S. maltophilia*	94 (42.3)	29 (41.4)	0.963 (0.558–1.661)	0.893	0.874 (0.473–1.615)	0.667
Source control measures	50 (22.5)	8 (11.4)	0.444 (0.199–0.989)	0.057	0.548 (0.175–1.721)	0.303

^*a*^ APACHE II, Acute Physiology and Chronic Health Evaluation; CI, confidence interval; OR, odds ratio.

### Secondary outcomes

No significant differences were observed between the CT and MT groups in terms of secondary outcomes, including in-hospital mortality, clinical response, microbiological eradication, and emergence of non-susceptible *S. maltophilia* isolates (Table 3). Among the 282 patients with follow-up cultures, the rates of non-susceptible *S. maltophilia* occurrence to levofloxacin, SXT, and minocycline were similar between the groups (Table 3). Resistance patterns are detailed in Table S8, with the most common being concurrent non-susceptibility to levofloxacin and SXT (*n* = 21), followed by levofloxacin only (*n* = 12), and SXT-only (*n* = 11) resistance. Further clinical information for those patients was shown in Tables S9 and S10. ADRs occurred in 38 patients (13.0%) and did not differ significantly between groups (18.9% vs 12.2%, *P* = 0.293; Table 4). Hyperkalemia (*n* = 25) was the most common complication. Most events were moderate (*n* = 30) or mild (*n* = 8) according to the Hartwig Severity Assessment Scale. Seven patients (2.3%) discontinued antibiotics because of adverse events.

**TABLE 3:** Clinical and microbiologic outcomes in patients with *S. maltophilia* bacteremia treated with CT vs MT^*d*^

Variables, *n* (%)	Total (*n* = 292)	CT (*n* = 37)	MT (*n* = 255)	*P* value
30-day mortality	70 (24.0)	4 (10.8)	66 (25.9)	0.045
In-hospital mortality	108 (37.0)	14 (37.8)	94 (36.9)	0.909
Clinical response after EOT	210 (71.9)	27 (73.0)	183 (71.8)	0.879
Microbiological eradication (*n* = 265)^*a*^	247 (93.2)	35 (94.6)	212 (93.0)	1.000
Recurrence bacteremia (*n* = 247)^*b*^	20 (8.1)	0 (0)	20 (9.4)	0.087
Non-susceptible *S. maltophilia* isolation (*n* = 282)^*c*^	47 (16.0)	9 (24.3)	36 (14.9)	0.136

^*a*^ 265 patients with follow-up blood cultures available are included in the analysis.

^*b*^ 247 patients with documented microbiologic eradication from blood are included for bacteremia recurrence analysis.

^*c*^ 282 patients with follow-up cultured *S. maltophilia* are included for analysis.

^*d*^ CT: combination therapy; MT: monotherapy; LOS: length of stay; EOT: end of therapy.

**TABLE 4:** Safety data among patients with *S. maltophilia* bacteremia who received CT vs MT^*c*^

Factors, *n* (%)	Total (*n* = 292)	CT (*n* = 37)	MT (*n* = 255)	*P* value
Any adverse reaction	38 (13.0)	7 (18.9)	31 (12.2)	0.293
Skin rash	3	0	3	
Hyperkalemia^*a*^	24	2	22	
Agranulocytosis^*b*^	2	0	2	
Diarrhea	3	3	0	
Hepatitis	5	1	4	
Hyperkalemia + Diarrhea + Hepatitis	1	1	0	
Discontinued antibiotic use due to adverse drug reactions	7 (2.4)	0 (0)	7 (2.7)	1.000

^*a*^ Serum potassium level >5.5 mmol/L.

^*b*^ Absolute neutrophil count <1,500/μL.

^*c*^ CT, combination therapy; MT, monotherapy.

## DISCUSSION

This study compared the clinical, microbiological, and safety outcomes of patients receiving CT vs MT for monomicrobial *S. maltophilia* bacteremia. The results showed that patients receiving CT had a lower risk of 30-day mortality than those receiving MT, both in the crude and adjusted analyses. The rates of bacteremia recurrence, *S. maltophilia* resistance development, and ADR were comparable between the CT and MT groups.

Several studies comparing the clinical outcomes of CT and MT for *S. maltophilia* pneumonia or bacteremia have reported conflicting results (12–21). Regarding *S. maltophilia* pneumonia, four relevant studies were identified (12–15). Two studies found no significant difference in 30-day mortality (12, 13), whereas Shah et al. reported higher mortality with CT (14). Another study by Chen et al. demonstrated significantly lower odds of 30-day mortality associated with CT in two subgroups: immunocompromised patients and those with APACHE II scores ≥15 (15). The diagnosis of *S. maltophilia* pneumonia in these studies was based on clinical symptoms, imaging findings, laboratory data, and respiratory cultures. However, due to the low virulence of *S. maltophilia*, distinguishing between colonization and true infection is difficult, complicating the evaluation of treatment efficacy (30, 31). Moreover, *S. maltophilia* was co-isolated with other respiratory pathogens in 34.3%–54% of cases in these studies, most commonly *Pseudomonas aeruginosa*. In polymicrobial pneumonia, co-pathogens may also affect the outcomes (32, 33). A recent study of deep-seated *S. maltophilia* infections found that levofloxacin-based combination therapy was associated with reduced odds of clinical failure in adjusted analyses, suggesting a potential benefit of combination therapy. However, other coinfections commonly observed in *S. maltophilia* infections were not adjusted for and may have introduced bias (16). Three studies involving patients with *S. maltophilia* bacteremia were noted (17, 19, 21). One study involving patients with cancer found no association between CT and improved outcomes (21). The study by Muder et al. reported a benefit of CT in immunocompromised patients, whereas the study by Osawa et al. showed opposite results (17, 19). Given the limited number of bacteremia cases, adjustment for certain mortality-related confounders was not feasible, and polymicrobial bacteremia was not consistently excluded, which may have biased the findings. Finally, none of the reported studies on patients with pneumonia or bacteremia evaluated the appropriateness of the dosing regimens used.

To address the limitations of previous studies, the current study included only patients with monomicrobial *S. maltophilia* bacteremia, which clearly defines true invasive infection and excludes the potential influence of co-pathogens in the blood. Moreover, several confounding factors related to mortality were adjusted for, including antibiotic treatment and coinfections other than bacteremia. Treatment duration, which showed a significant difference between the CT and MT groups in the current study (Table 1), was not included in the multivariable analysis due to the risk of survival bias. In the adjusted multivariable regression models and across several additional sensitivity analyses, CT demonstrated a consistent trend toward improved 30-day mortality (Table 2; Table S5 to S7). Subgroup analysis also showed that the beneficial effects were similar across different infection site sources (Fig. 3). CT can provide broader spectrum coverage than MT, thereby increasing the rate of appropriate immediate antibiotic use, as observed in the current study, which is a factor crucial for improved outcomes in patients with *S. maltophilia* bacteremia (34). In addition, the enhanced efficacy of CT in *in vitro* studies may play a role (8, 9). Nevertheless, not all antibiotic combinations have demonstrated similar effects in previous studies, and even the same combination has shown varying antimicrobial activity against different *S. maltophilia* isolates (8, 9). In the current study, levofloxacin with SXT was the most commonly used combination regimen; however, synergistic antimicrobial activity was observed in only approximately 24% of tested isolates in an *in vitro* study (21).

We also compared the occurrence of adverse drug reactions (ADRs) and the risk of developing resistance between the CT and MT groups. The overall ADR rate was 13.0%, which is consistent with previous studies (35). All reported ADRs were classified as mild-to-moderate according to the Hartwig Severity Assessment Scale. Only seven patients (2.4%) discontinued antibiotic treatment because of adverse events. SXT-related hyperkalemia was the most frequently reported ADR and the leading cause of treatment discontinuation. Consistent with our findings, a recent study by Chen et al. reported that SXT-related hyperkalemia was a major adverse drug reaction during the treatment of *S. maltophilia* bacteremia, particularly at higher doses. Notably, that study observed no significant difference in 30-day mortality between patients receiving high- vs low-dose SXT therapy (28). Further studies are warranted to define the optimal SXT dosing strategy for the treatment of *S. maltophilia* infections; however, clinicians should remain vigilant for this potentially life-threatening ADR. Hepatitis associated with levofloxacin was infrequent and mild in severity; therefore, none of the patients discontinued levofloxacin because of hepatitis. Owing to the favorable safety profile and tolerability of levofloxacin, the combination regimen of levofloxacin and SXT, which was the main CT regimen in this study, did not increase the risk of ADRs or treatment discontinuation.

*S. maltophilia* isolates with concurrent non-susceptibility to SXT and levofloxacin were the most frequently observed resistant phenotypes among follow-up cultures after antibiotic use. This resistance mechanism may be attributed to the overexpression of SmeVWX or SmeDEF, which can lead to concomitant SXT and levofloxacin resistance when either agent is used, as previously demonstrated (36). Several *in vitro* studies have shown that acquired resistance to *S. maltophilia* may occur after the use of individual agents (36, 37). Therefore, CT for *S. maltophilia* infection is suggested not only for its potentially enhanced antimicrobial efficacy but also for the possible prevention of resistance emergence. Our study evaluated the effect of CT on preventing resistance development; however, the results did not demonstrate a protective effect against the emergence of resistant strains. Because only 37 patients received CT in the current study, further research involving a larger cohort of CT cases is needed to validate these findings.

This study has some important limitations. First, its retrospective design is inherently susceptible to selection and recall biases. Although major confounders influencing the outcomes were adjusted using multivariable models, unmeasured residual confounders that could affect the analysis remain a concern (29–34). Second, the relatively small sample size of the CT group (*n* = 37) may have reduced statistical power and affected the interpretation of the results. The limited sample size also precluded subgroup analyses based on different combination regimens or specific host populations. Third, suboptimal antibiotic dosing for the treatment of *S. maltophilia* infection (only 123 patients [42.1%] in both treatment groups received optimal doses) may have influenced treatment efficacy although this association was not identified in the logistic regression analysis. Fourth, ADRs were assessed through a medical chart review. Given that 211 cases (72.3%) involved ICU admission, multiple comorbidities, and concurrent medical treatments in addition to receiving antibiotics for *S. maltophilia* infection, it was often difficult to attribute adverse reactions solely to antibiotic therapy. This may have introduced bias in the estimates. Fifth, the high rate of bacterial coinfections in this cohort, including 23.6% caused by carbapenem-resistant organisms, limited our ability to isolate the specific effect of CT vs MT on outcomes of *S. maltophilia* infection. Sixth, the exclusion of *S. maltophilia* treatment with other potentially active agents, such as colistin, may limit the generalizability of our findings to centers where these additional agents are commonly used. Finally, the single-center design of this study limits the generalizability of our results to other institutions or geographic regions.

### Conclusion

In this observational study, CT for *S. maltophilia* bacteremia was associated with more favorable outcomes and comparable rates of adverse drug reactions and resistance emergence. Our clinical data support the current recommendations advocating CT for the treatment of *S. maltophilia* infections. However, given the limitations of this study, further evidence from randomized studies with larger sample sizes is required to confirm these findings.

## SUPPLEMENTAL MATERIAL

Fig. S1 aac.01297-25-s0001.tiff Non-susceptibility trend of *Stenotrophomonas maltophilia* isolates to SXT and levofloxacin over the study period.

Supplemental material aac.01297-25-s0002.pdf Fig. S1 legend.

Supplemental tables aac.01297-25-s0003.doc Tables S1 to S10.

ASM does not own the copyrights to Supplemental Material that may be linked to, or accessed through, an article. The authors have granted ASM a non-exclusive, world-wide license to publish the Supplemental Material files. Please contact the corresponding author directly for reuse.
