# Supplementary figures and images for: Treatment outcomes of combination versus monotherapy in Stenotrophomonas maltophilia bacteremia: a retrospective single-center analysis
Source: Antimicrob Agents Chemother. 2026 Apr 30;70(6):e01297-25. doi: 10.1128/aac.01297-25 (PMC13231920; doi:10.1128/aac.01297-25)

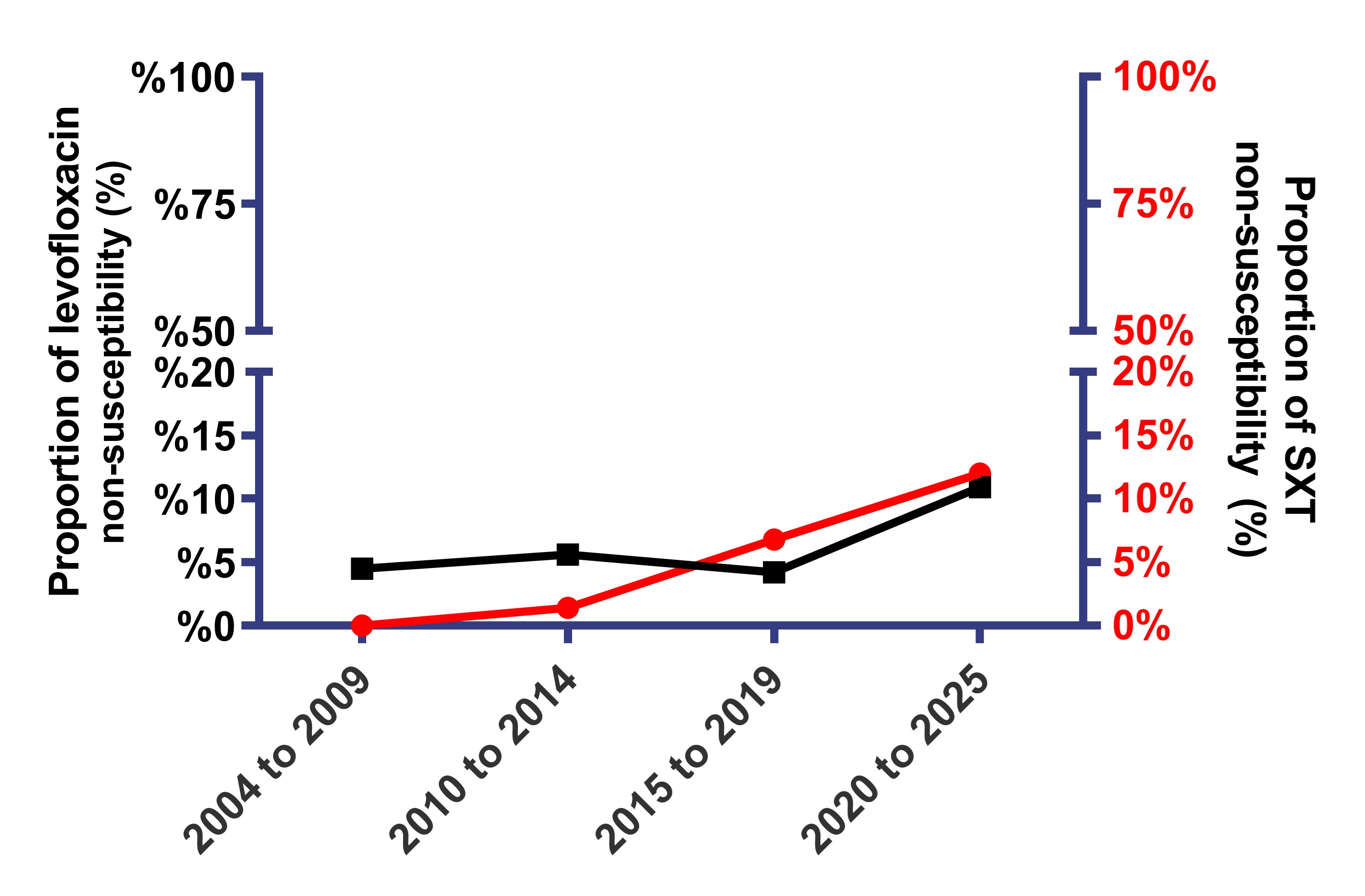

Supplement: Fig. S1 — Non-susceptibility trend of Stenotrophomonas maltophilia isolates to SXT and levofloxacin over the study period. [file aac.01297-25-s0001.tiff]
