# Supplementary material for: Treatment outcomes of combination versus monotherapy in Stenotrophomonas maltophilia bacteremia: a retrospective single-center analysis
Source: Antimicrob Agents Chemother. 2026 Apr 30;70(6):e01297-25. doi: 10.1128/aac.01297-25 (PMC13231920; doi:10.1128/aac.01297-25)
Supplement: Supplemental material — Fig. S1 legend. [file aac.01297-25-s0002.pdf]

Supplementary Figure 1

Non-susceptibility trend of Stenotrophomonas maltophilia isolates to SXT and levofloxacin over the study period. SXT, trimethoprim/sulfamethoxazole
