# Supplementary material for: Treatment outcomes of combination versus monotherapy in Stenotrophomonas maltophilia bacteremia: a retrospective single-center analysis
Source: Antimicrob Agents Chemother. 2026 Apr 30;70(6):e01297-25. doi: 10.1128/aac.01297-25 (PMC13231920; doi:10.1128/aac.01297-25)
Supplement: Supplemental tables — Tables S1 to S10. [file aac.01297-25-s0003.doc]

Supplementary Table 1. Different types of bacterial co-infection episodes among patients with *Stenotrophomonas maltophilia* bacteremia

| Infection, n (%) | Total (n = 292) | CT (n = 37) | MT (n = 255) |
| --- | --- | --- | --- |
| **Pneumonia** | 101 (34.5) | 12 (32.4) | 89 (34.9) |
| Intra-abdominal infection | 3 (1.0) | 0 (0) | 3 (1.1) |
| Urinary tract infection | 18 (6.1) | 2 (5.4) | 16 (6.2) |
| Skin and soft tissue infection | 14 (4.7) | 1 (2.7) | 13 (5.0) |

CT, combination therapy; MT, monotherapy.

Supplementary Table 2. Detected pathogens in patients with *S. maltophilia* bacteremia with bacterial co-infections

| Pathogen, n (%) | Total (n = 292) | CT (n = 37) | MT (n = 255) |
| --- | --- | --- | --- |
| Cultured pathogens from pneumonia |  |  |  |
| *Acinetobacter* spp. | 51(17.4) | 6 (16) | 45 (17.6) |
| *Pseudomonas aeruginosa* | 22 (7.5) | 4 (10.8) | 18 (7.0) |
| *Klebsiella pneumoniae* | 17 (5.8) | 4 (10.8) | 13 (5.0) |
| *Staphylococcus aureus* | 13 (4.4) | 0 (0) | 13 (5.0) |
| *Elizabethkingia anophelis* | 10 (3.4) | 2 (5.4) | 8 (3.1) |
| *Chryseobacterium indologenes* | 7 (2.3) | 2 (5.4) | 5 (1.9) |
| *Enterobacter cloacae* | 7 (2.3) | 1 (2.7) | 6 (2.3) |
| *Chryseobacterium meningosepticus* | 6 (2.0) | 0 (0) | 6 (2.3) |
| *Serratia marcescens* | 4 (1.3) | 1 (2.7) | 3 (1.1) |
| *Achromobacter xylosoxidans* | 3 (1.0) | 0 (0) | 3 (1.1) |
| *Delftia acidovorans* | 3 (1.0) | 0 (0) | 3 (1.1) |
| *Proteus mirabilis* | 3 (1.0) | 0 (0) | 3 (1.1) |
| *Sphingomonas paucimobilis* | 3 (1.0) | 2 (5.4) | 1 (0.3 ) |
| *Streptococcus agalactiae* | 2 (0.6) | 1 (2.7) | 1 (0.3) |
| *Burkholderia cepacia* | 1 (0.3) | 0 (0) | 1 (0.3) |
| *Citrobacter freundii* | 1 (0.3) | 0 (0) | 1 (0.3) |
| *Citrobacter diversus* | 1 (0.3) | 0 (0) | 1 (0.3) |
| *Escherichia coli* | 1 (0.3) | 0 (0) | 1 (0.3) |
| *Pseudomona putida* | 1 (0.3) | 0 (0) | 1 (0.3) |
| Cultured pathogens from urinary tract infection |  |  |  |
| *Enterococcus faecium* | 7 (2.3) | 0 (0) | 7 (2.7) |
| *Escherichia coli* | 5 (1.7) | 1 (2.7) | 4 (1.5) |
| *Pseudomonas aeruginosa* | 2 (0.6) | 0 (0) | 2 (0.7) |
| *Acinetobacter baumannii* spp. | 1 (0.3) | 0 (0) | 1 (0.3) |
| *Enterococcus faecalis* | 1 (0.3) | 1 (2.7) | 0 (0) |
| *Enterococcus* spp. | 1 (0.3) | 0 (0) | 1 (0.3) |
| *Klebsiella pneumoniae* | 1 (0.3) | 0 (0) | 1 (0.3) |
| *Morganella morganii* | 1 (0.3) | 1 (2.7) | 0 (0) |
| *Proteus mirabilis* | 1 (0.3) | 1 (2.7) | 0 (0) |
| *Staphylococcus aureus* | 1 (0.3) | 0 (0) | 1 (0.3) |
| Cultured pathogens from skin and soft tissue infection |  |  |  |
| *Acinetobacter* spp. | 5 (1.7) | 1 (2.7) | 4 (1.5) |
| *Enterococcus faecalis* | 2 (0.6) | 1 (2.7) | 1 (0.3) |
| *Bacteroides* spp. | 2 (0.6) | 0 (0) | 2 (0.7) |
| *Enterococcus faecium* | 2 (0.6) | 0 (0) | 2 (0.7) |
| *Staphylococcus aures* | 2 (0.6) | 0 (0) | 2 (0.7) |
| *Chryseobacterium indologenes* | 1 (0.3) | 0 (0) | 1 (0.3) |
| *Enterococcus* spp. | 1 (0.3) | 1 (2.7) | 0 (0) |
| *Escherichia coli* | 1 (0.3) | 0 (0) | 1 (0.3) |
| *Enterobacter cloacae* | 1 (0.3) | 0 (0) | 1 (0.3) |
| *Proteus mirabilis* | 1 (0.3) | 0 (0) | 1 (0.3) |
| Cultured pathogen from intra-abdominal infection |  |  |  |
| *Enterococcus faecium* | 2 (0.6) | 0 (0) | 2 (0.7) |
| *Bacteroides* spp. | 1 (0.3) | 0 (0) | 1 (0.3) |
| *Escherichia coli* | 1 (0.3) | 0 (0) | 1 (0.3) |
| *Fusobacterium mortiferum* | 1 (0.3) | 0 (0) | 1 (0.3) |
| *Proteus mirabilis* | 1 (0.3) | 0 (0) | 1 (0.3) |
| *Streptococcus* spp. | 1 (0.3) | 0 (0) | 1 (0.3) |

CT, combination therapy; MT, monotherapy.

Supplementary Table 3. Carbapenem-resistant bacteria isolated from patients receiving combination therapy versus monotherapy.

| Pathogen, n (%) | Total (n = 292) | CT (n = 37) | MT (n = 255) |
| --- | --- | --- | --- |
| *Acinetobacter,* spp. | 23 (7.8) | 4 (10.8) | 19 (7.4) |
| *Pseudomonas aeruginosa* | 8 (2.7) | 2 (5.4) | 6 (2.3) |
| *Elizabethkingia anopheles* | 6 (2.0) | 2 (5.4) | 4 (1.5) |
| *Chryseobacterium indologenes* | 3 (1.0) | 2 (5.4) | 1 (0.3) |

CT, combination therapy; MT, monotherapy.

Supplementary Table 4. Concurrent use of antibiotics other than SXT, levofloxacin, and minocycline in patients receiving CT versus MT.

|  | CT (n = 37) | MT (n = 255) | *P* value |
| --- | --- | --- | --- |
| Cephalosporins | 8 (21.6) | 48 (18.8) | 0.686 |
| Ceftriaxone | 1 | 2 |  |
| Flomoxef | 0 | 2 |  |
| Ceftazidime | 5 | 13 |  |
| Cefepime | 2 | 26 |  |
| Cefpirome | 0 | 5 |  |
| Carbapenems | 8(21.6) | 79 (30.9) | 0.245 |
| Imipenem/cilastatin | 0 | 24 |  |
| Meropenem | 7 | 41 |  |
| Doripenem | 1 | 14 |  |
| β-lactam/lactamase inhibitors | 14 (37.8) | 60 (23.5) | 0.062 |
| Ampicillin/sulbactam | 0 | 4 |  |
| Ceftazidime/avibactam | 2 | 6 |  |
| Piperacillin/tazobactam | 5 | 21 |  |
| Cefoperazone/sulbactam | 7 | 29 |  |
| Aminoglycosides | 0 (0) | 8 (3.1) | 0.275 |
| Gentamycin | 0 | 1 |  |
| Amikacin | 0 | 7 |  |
| Polymyxines | 4 (10.8) | 26 (10.1) | 1.000 |
| Colistin E | 3 | 26 |  |
| Colistin B | 1 | 0 |  |
| Tetracyclines and glycylcyclines | 4 (10.8) | 26 (10.1) | 1.000 |
| Tigecycline | 4 | 24 |  |
| Doxycycline | 0 | 2 |  |
| Quinolones | 1 (2.7) | 11 (4.3) | 1.000 |
| Moxifloxcain | 1 | 1 |  |
| Ciprofloxacin | 0 | 10 |  |
| Glycopeptides | 7 (18.9) | 43 (16.8) | 0.756 |
| Vancomycin | 5 | 19 |  |
| Teicoplanin | 2 | 24 |  |
| Metronidazole | 1 (2.7) | 11 (4.3) | 1.000 |
| Fosfomycin | 2 (5.4) | 5 (1.9) | 0.218 |
| Daptomycin | 1 (2.7) | 10 (3.9) | 1.000 |
| Linezolid | 0 (0) | 7 (2.7) | 0.601 |

SXT, trimethoprim/sulfamethoxazole; CT, combination therapy; MT, monotherapy.

Supplementary Table 5. Sensitivity analysis using for 30-day in-hospital mortality after exclusing patients receiving combination therapy with

with colistin, tigecycline and other quinolones (MT group, n = 204; CT group, n = 37)

| **Variable** | **Univariable** | | **Multivariable** | |
| --- | --- | --- | --- | --- |
|  | **Unadjusted OR**  **(95% CI)** | ***P* value** | **Adjusted OR**  **(95% CI)** | ***P* value** |
| Age | 1.013 (0.993–1.032) | 0.198 |  |  |
| Male sex | 1.354 (0.704–2.603) | 0.363 |  |  |
| Charlson Comorbidity Index | 0.991 (0.874–1.125) | 0.893 |  |  |
| Hospital acquired | 0.587 (0.106–3.347) | 0.557 |  |  |
| **Source sites of bacteremia** |  |  |  |  |
| Respiratory tract | 2.362 (1.272–4.388) | 0.007 | 1.372 (0.635–2.966) | 0.421 |
| Central line-associated | 0.662 (0.324–1.349) | 0.256 |  |  |
| Unidentified origin | 0.296 (0.112–0.787) | 0.015 | 0.583 (0.180–1.887) | 0.368 |
| **Clinical conditions at bacteremia onset** |  |  |  |  |
| APACHE II score | 1.150 (1.091–1.214) | <0.001 | 1.134 (1.070–1.201) | <0.001 |
| Thrombocytopenia | 2.885 (1.514–5.498) | 0.001 | 2.119 (1.029–4.367) | 0.042 |
| Co-infection by other bacteria | 0.574 (0.296–1.112) | 0.100 |  |  |
| Co-infections by other carbapenem-resistant organisms | 0.870 (0.401–1.884) | 0.723 |  |  |
| **Antibiotic treatment** |  |  |  |  |
| Immediate effective antibiotic targeting *S. maltophilia* | 1.169 (0.559–2.442) | 0.678 | 1.079 (0.464–2.510) | 0.860 |
| Combination therapy for *S. maltophilia* | 0.354 (0.120–1.048) | 0.051 | 0.226 (0.067–0.761) | 0.016 |
| Appropriate antibiotic treatment dosage for *S. maltophilia* | 0.978 (0.533–1.794) | 0.942 | 0.948 (0.475–1.890) | 0.879 |
| **Source control measures** | 0.591 (0.247–1.414) | 0.237 |  |  |

CT, combination therapy; MT, monotherapy.

Supplementary Table 6. Sensitivity analysis using for 30-day in-hospital mortality after exclusing patients who did not have co-infection by

other bacteria (MT group, n = 153; CT group, n = 22).

| **Variable** | **Univariable** | | **Multivariable** | |
| --- | --- | --- | --- | --- |
|  | **Unadjusted OR**  **(95% CI)** | ***P* value** | **Adjusted OR**  **(95% CI)** | ***P* value** |
|  |  |  |  |  |
| Age | 1.002 (0.982–1.023) | 0.841 |  |  |
| Male sex | 1.518 (0.729–3.159) | 0.265 |  |  |
| Charlson Comorbidity Index | 1.008 (0.876–1.160) | 0.911 |  |  |
| Hospital acquired | 0.730 (0.065–8.245) | 0.799 |  |  |
| **Source sites of bacteremia** |  |  |  |  |
| Respiratory tract | 2.718 (1.368–5.399) | 0.004 | 1.668 (0.706–3.939) | 0.244 |
| Central line-associated | 0.655 (0.309–1.386) | 0.268 |  |  |
| Unidentified origin | 0.257 (0.086–0.770) | 0.015 | 0.556 (0.155–1.993) | 0.368 |
| **Clinical conditions at bacteremia onset** |  |  |  |  |
| APACHE II score | 1.124 (1.064–1.189) | <0.001 | 1.092 (1.027–1.162) | 0.005 |
| Thrombocytopenia | 3.500 (1.693–7.237) | <0.001 | 2.760 (1.217–6.259) | 0.015 |
| **Antibiotic treatment** |  |  |  |  |
| Immediate effective antibiotic targeting *S. maltophilia* | 0.988 (0.406–2.403) | 0.979 | 0.928 (0.331–2.607) | 0.888 |
| Combination therapy for *S. maltophilia* | 0.391 (0.110–1.389) | 0.146 | 0.261 (0.064–1.062) | 0.061 |
| Appropriate antibiotic treatment dosage for *S. maltophilia* | 0.685 (0.341–1.376) | 0.288 | 0.702 (0.317–1.556) | 0.383 |
| **Source control measures** | 0.545 (0.232–1.281) | 0.164 |  |  |

CT, combination therapy; MT, monotherapy; APACHE II, Acute Physiology and Chronic Health Evaluation; CI, confidence interval; OR, odds ratio

Supplementary Table 7. Sensitivity analysis using for 30-day in-hospital mortality after exclusing patients who did not have co-infection by

*Acinetobacter* spp. (MT group, n = 208; CT group, n = 30).

| **Variable** | **Univariable** | | **Multivariable** | |
| --- | --- | --- | --- | --- |
|  | **Unadjusted OR**  **(95% CI)** | ***P* value** | **Adjusted OR**  **(95% CI)** | ***P* value** |
| Age | 1.008 (0.990–1.028) | 0.382 |  |  |
| Male sex | 1.043 (0.561–1.938) | 0.895 |  |  |
| Charlson Comorbidity Index | 0.978 (0.863–1.107) | 0.722 |  |  |
| Hospital acquired | 0.497 (0.081–3.049) | 0.450 |  |  |
| **Source sites of bacteremia** |  |  |  |  |
| Respiratory tract | 2.484 (1.358–4.545) | 0.003 | 1.738 (0.818–3.693) | 0.151 |
| Central line-associated | 0.699 (0.355–1.377) | 0.301 |  |  |
| Unidentified origin | 0.269 (0.101–0.713) | 0.008 | 0.591 (0.186–1.876) | 0.372 |
| **Clinical conditions at bacteremia onset** |  |  |  |  |
| APACHE II score | 1.128 (1.073–1.185) | <0.001 | 1.100 (1.042–1.161) | <0.001 |
| Thrombocytopenia | 3.465 (1.843–6.514) | <0.001 | 2.567 (1.267–5.199) | 0.009 |
| Co-infection by other bacteria | 0.708 (0.353–1.420) | 0.331 |  |  |
| Co-infections by other carbapenem-resistant organisms | 1.091 (0.458–2.599) | 0.844 |  |  |
| **Antibiotic treatment** |  |  |  |  |
| Immediate effective antibiotic targeting *S. maltophilia* | 1.059 (0.508–2.208) | 0.879 | 1.024 (0.442–2.373) | 0.956 |
| Combination therapy for *S. maltophilia* | 0.418 (0.140–1.250) | 0.118 | 0.273 (0.081–0.925) | 0.037 |
| Appropriate antibiotic treatment dosage for *S. maltophilia* | 0.795 (0.435–1.454) | 0.456 | 0.734 (0.372–1.448) | 0.373 |
| **Source control measures** | 0.531 (0.233–1.209) | 0.132 |  |  |

CT, combination therapy; MT, monotherapy; APACHE II, Acute Physiology and Chronic Health Evaluation; CI, confidence interval; OR, odds ratio

Supplementary Table 8. Development of antibiotic resistance from follow-up cultured *S. maltophilia* in patients treated with CT versus MT^a^.

| Antibiotic nonsusceptibility profiles, n (%) |  | Total (n = 282) | CT (n = 37) | MT (n = 245) |
| --- | --- | --- | --- | --- |
| SXT only |  | 12 (4.3) | 0 (0) | 12 (4.9) |
| Levofloxacin only |  | 11 (3.9) | 2(5.4) | 9 (3.7) |
| Levofloxacin + SXT |  | 21 (7.4) | 6 (16.2) | 15 (6.1) |
| Levofloxacin + SXT +minocycline |  | 1 (0.3) | 1 (2.7) | 0 (0) |

SXT, trimethoprim/sulfamethoxazole; CT, combination therapy; MT, monotherapy.

^a^A total of 282 patients with follow-up cultured *S. maltophilia* were included.

Supplementary Table 9. Clinical information of patients treated with combination therapy (CT) in whom subsequent non-susceptible

*S. maltophilia isolates were identified*

| Non-susceptibility profile | Case number | Age, yr | Weight, kg | Renal function at baseline | Starting daily treatment dosage | Elapsed time between bacteremia and treatment, day |
| --- | --- | --- | --- | --- | --- | --- |
| Levofloxacin only | 425 | 79 | 53 | 8.67 | SXT 0.64mg/kg^a^ + levofloxacin 250mg | 3 |
|  | 450 | 77 | 70 | 26.63 | Minocycline 200mg + levofloxacin 250mg | 1 |
| Levofloxacin + SXT | 347 | 40 | 67 | 15.25 | SXT 2.71 mg/kg + levofloxacin 250 mg | 0 |
|  | 353 | 25 | 70 | 53.24 | SXT 2.29 mg/kg + levofloxacin 250 mg | 2 |
|  | 378 | 73 | 73 | 226.43 | SXT 6.58 mg/kg + levofloxacin 750 mg | 0 |
|  | 384 | 72 | 60 | 40.13 | SXT 16 mg/kg + levofloxacin 250 mg | 12 |
|  | 413 | 66 | 50 | 19.03 | SXT 8 mg/kg + levofloxacin 250 mg | 5 |
|  | 428 | 80 | 52 | 22.80 | Minocycline 200mg + levofloxacin 250 mg | 4 |
| Levofloxacin + SXT + minocycline | 418 | 88 | 55 | 10.89 | SXT3 mg/kg + levofloxacin 250 mg | 1 |

SXT, trimethoprim/sulfamethoxazole; CT, combination therapy.

^a^Based upon the trimethoprim component.

Supplementary Table 10. Clinical information of patients treated with monotherapy (MT) in whom subsequent non-susceptible

*S. maltophilia isolates were identified*

| Non-susceptibility profile | Case number | Age, yr | Weight, kg | Bseline creatinine level, mg/dL | Daily Treatment Dosage^a^ | Elapsed time between bacteremia and treatment, day |
| --- | --- | --- | --- | --- | --- | --- |
| SXT only | 13 | 88 | 70 | 1.2 | SXT 2.85 mg/kg | 7 |
|  | 20 | 58 | 43 | 0.5 | SXT 22 mg/kg | 3 |
|  | 37 | 78 | 48 | 1.4 | SXT 6.67 mg/kg | 4 |
|  | 137 | 75 | 65 | 1.3 | SXT 2.6 mg/kg | 4 |
|  | 155 | 78 | 68 | 5.2 | SXT 7.05 mg/kg | 9 |
|  | 159 | 79 | 58 | 0.5 | SXT 5.5 mg/kg | 5 |
|  | 161 | 84 | 55 | 3.1 | SXT 3.08 mg/kg | 7 |
|  | 168 | 64 | 55 | 0.4 | SXT 5.8 mg/kg | 3 |
|  | 171 | 82 | 50 | 0.9 | SXT 6.4 mg/kg | 5 |
|  | 269 | 80 | 40 | 0.4 | Levofloxacin 500 mg | 0 |
|  | 278 | 89 | 70 | 2.2 | SXT 6.8 mg/kg | 8 |
|  | 380 | 67 | 50 | 0.5 | SXT 6.4 mg/kg | 10 |
| Levofloxacin only | 220 | 81 | 87 | 0.5 | Levofloxacin 750 mg | 6 |
|  | 223 | 65 | 40 | 0.6 | Levofloxacin 375 mg | 5 |
|  | 251 | 73 | 60 | 0.7 | Levofloxacin 250 mg | 0 |
|  | 252 | 75 | 60 | 0.8 | Levofloxacin 750 mg | 4 |
|  | 281 | 79 | 65 | 0.9 | Levofloxacin 750 mg | 12 |
|  | 329 | 64 | 51 | 3 | Levofloxacin 750 mg | 4 |
|  | 345 | 90 | 51 | 1.3 | Levofloxacin 750 mg | 5 |
|  | 400 | 47 | 55 | 1.5 | Levofloxacin 750 mg | 5 |
|  | 443 | 72 | 54 | 3.6 | Levofloxacin 250 mg | 6 |
| Levofloxacin + SXT | 204 | 62 | 59 | 1.1 | Levofloxacin 750 mg | 0 |
|  | 259 | 64 | 45 | 0.7 | Levofloxacin 750 mg | 1 |
|  | 262 | 71 | 55.4 | 5.6 | Levofloxacin 250 mg | 7 |
|  | 266 | 55 | 82 | 2.2 | Levofloxacin 250 mg | 1 |
|  | 270 | 73 | 52 | 5.6 | Levofloxacin 250 mg | 0 |
|  | 271 | 52 | 55 | 1 | Levofloxacin 750 mg | 5 |
|  | 277 | 78 | 80 | 2.3 | Levofloxacin 250 mg | 3 |
|  | 297 | 77 | 64 | 9.5 | Levofloxacin 250 mg | 5 |
|  | 309 | 89 | 74 | 0.5 | Levofloxacin 750 mg | 0 |
|  | 364 | 39 | 61 | 0.6 | Levofloxacin 750 mg | 0 |
|  | 373 | 95 | 66 | 2.5 | Levofloxacin 500 mg | 3 |
|  | 381 | 52 | 60 | 0.7 | Levofloxacin 750 mg | 0 |
|  | 403 | 75 | 67 | 4.4 | Levofloxacin 250 mg | 0 |
|  | 438 | 81 | 56 | 0.7 | Levofloxacin 750 mg | 1 |
|  | 469 | 73 | 59.5 | 2.1 | Levofloxacin 250 mg | 4 |

SXT, trimethoprim/sulfamethoxazole; MT, monotherapy

^a^Based upon the trimethoprim component.
